# Editorial: Diverse roles of MAP4K4 in MAP kinase signaling and its implication for cancer therapeutics

**DOI:** 10.3389/fonc.2023.1248808

**Published:** 2023-07-19

**Authors:** Ruchi Roy, Sunil Kumar Singh, Ajay Rana

**Affiliations:** ^1^UICentre for Drug Discovery, The University of Illinois at Chicago, Chicago, IL, United States; ^2^Department of Surgery, Division of Surgical Oncology, The University of Illinois at Chicago, Chicago, IL, United States

**Keywords:** mitogen activate protein kinase, MAP4K4, oncogenes, cancer therapeutic, immunotherapy

Mitogen-activated protein kinases (MAPK) signaling represents essential signaling cascades controlling cellular metabolism, motility, cell death, and survival in response to intracellular or extracellular stimuli. MAPKs, a family of protein kinases, are activated through the phosphorylation of their own dual serine and threonine residues within the catalytic domains, leading to the phosphorylation of their substrates to activate or inhibit their functions. MAPK is a complex signaling cascade involving multiple tiers of kinases, transmitting signals upon sequential phosphorylation by the upstream kinases and ultimately leading to cellular fate.

One of the upstream integrators of MAP kinases is Mitogen-activated protein 4 kinase 4 (MAP4K4), a serine/threonine protein kinase implicated in various pathological conditions that play a pivotal role in embryonic cell development, cancer cells transformation, invasiveness, adhesion, migration, and inflammation. Moreover, its dysregulation contributes to the development of metabolic diseases, cardiovascular diseases, type 2 diabetes, anti-viral immunity, and initiation and progression of cancer. MAP4K4 plays a critical role in MLK3-mediated MAP Kinase signaling and has been implicated in several types of cancers, including glioblastoma, colon, prostate, and pancreatic cancers. However, reports on the roles of MAP4K4 in cancers are remarkably insignificant, which prompted us to fathom this Research Topic highlighting the significance of MAP4K4 and its potential to be a viable target in cancers. The available data on the role of MAP4K4 in cancers suggest its overexpression in multiple organs, e.g., the brain, heart, and testis, and its expression vary in a tissue-specific manner. Although MAP4K4 has been implicated in controlling tumor growth and progression, there are still many challenges ahead that demand in-depth studies to consider MAP4K4 as an actionable cancer target before taking its inhibitors for clinical trials.

Four research articles in this Research Topic cover the dynamic role of MAP kinases, including MAP4K4, in cancer. Findings of additional effectors of MAP4K4 apart from its role as an activator of the c-Jun N-terminal kinase (JNK) have expanded its role in various cellular functions. Review articles in this Research Topic highlighted its role in cancer initiation and progression by controlling proliferation and cell motility. The review article by Jovanovic et al. emphasizes the significance of MAP4K4 in neuronal cell death in neurodegenerative diseases or cell dissemination in malignant tumors causing damage to the early blood-brain barrier damage. Its expression is highest in testicular and pancreatic tissue and nervous system cells to promote vascular permeability and induce leukocyte adhesion and infiltration in the tumor microenvironment, which could represent a biomarker associated with poor clinical prognosis (Jovanovic et al.; González-Montero et al.). Another mini-review on “MAP4K4 and cancer: ready for the main stage” by González-Montero et al. describes how inhibiting MAP4K4 function using RNA interference-based knockdown (miR) techniques reduces tumor growth and invasion, and this approach suggests a promising therapeutic value of MAP4K4 in many types of cancers. They discussed how deletion of MAP4K4 can be detrimental and cause alteration in embryonic development and impaired cell migration, proliferation, and growth. The authors draw attention to GNE-495, a specific inhibitor of MAP4K4 that can reduce tumor-promoting activities associated with MLK3 phosphorylation. Pharmacological inhibition of MAP4K4 with GNE-495 inhibited pancreatic cell growth and migration and could be used as a novel agent for cancer treatment in the future. They also pointed out the importance of using miR-141 to inhibit MAP4K4 in colorectal cancer to increase tumor cell chemosensitivity and diminish their proliferation, invasion, and migration.

Thus, collectively, it is reasonable to ponder that MAP4K4 plays an important role in cell proliferation, transformation, invasion, adhesion, inflammation, stress responses, and cell migration through the c-Jun NH2-terminal kinase. Moreover, some reports suggest that different isoforms of MAP4K4 have distinct functions in various organs (1, 2). The consequence of this variation led it to control many physiological and pathophysiological activities (3–5). Its overexpression affects the tumor size and the patient’s survival. Several reports suggested its role as a proinflammatory kinase that enhances endothelial permeability, causing atherosclerosis (6). Inhibition of MAP4K4 hinders tumorigenesis by inducing cell cycle arrest and apoptosis, supporting the opinion that MAP4K4 could serve as a target in cancer.

A research article by Ren et al. on one of the important MAPK known as MAPK-activated protein kinase 3 (MAPKAPK3/MK3), which is also a serine/threonine protein kinase controls tumor progression and immunity in glioma and can serve as a prognostic marker. The results suggest that MK3 could be a promising target for glioma immunotherapy. They also reported MK3 is overexpressed in glioma and helps in immune infiltrations because of its functional relevance to regulating NK (Natural Killer cells) cell cytotoxicity and CD4 T-cell development, which could be utilized as a promising target for glioma immunotherapy. Their results suggest a positive correlation between MK3 expression and the majority of chemokines and chemokine receptors, such as CCL (2, 5, 8, 18, 22), CXCL12, CCR (1, 2, 5), CXCR2, CXCR4, and CX3CR1. Thus, their findings highlighted the critical role of MK3 in immune infiltrations in glioma and could be used for possible immunotherapy.

Another research article on how photodynamic therapy (PDT) can be applied against cutaneous squamous cell carcinoma (cSCC) by Zhao et al. has elucidated the role of the MAPK pathway in cell cycle alternation in cSCC. In this study, the GEO database has been used for expressing mRNA profile data sets GSE98767, GSE45216, and GSE84758 to perform correlation analysis to evaluate the relationship between cSCC-PDT-related genes and the MAPK pathway. They identified four cSCC-PDT-related genes, such as DUSP6, EFNB2, DNAJB1, and CCNL1, which were found to be upregulated in cSCC or LC50 PDT-protocol treatment and could be used as a potential PDT target gene in cSCC treatment inhibits MAPK promoter.

Although further in-depth studies are still necessary to fully understand the association of MAPKs, especially MAP4K4, with other cellular mediators and identify potential therapeutic targets, multiple research data and this Research Topic articles findings have recognized MAPKs and MAP4K4 as a viable therapeutic targets to treat several conditions such as vascular inflammation, atherosclerosis, and cancer.

## Author contributions

RR, SS, and AR wrote and edited the final draft of this editorial. All authors contributed to the article and approved the submitted version.
